# The Impact of FLT3 Mutations on the Development of Acute Myeloid Leukemias

**DOI:** 10.1155/2013/275760

**Published:** 2013-07-09

**Authors:** Ugo Testa, Elvira Pelosi

**Affiliations:** Department of Hematology, Oncology and Molecular Medicine, Upper Health Institute, Viale Regina Elena 299, 00161 Rome, Italy

## Abstract

The development of the genetic studies on acute myeloid leukemias (AMLs) has led to the identification of some recurrent genetic abnormalities. Their discovery was of fundamental importance not only for a better understanding of the molecular pathogenesis of AMLs, but also for the identification of new therapeutic targets. In this context, it is essential to identify AML-associated “driver” mutations, which have a causative role in leukemogenesis. Evidences accumulated during the last years indicate that activating internal tandem duplication mutations in FLT3 (FLT3-ITD), detected in about 20% of AMLs, represents driver mutations and valid therapeutic targets in AMLs. Furthermore, the screening of FLT3-ITD mutations has also considerably helped to improve the identification of more accurate prognostic criteria and of the therapeutic selection of patients.

## 1. Introduction

The FMS-like tyrosine (FLT3) gene encodes a class III receptor tyrosine kinase, sharing structural and sequence homologies with family members, including c-kit, c-FMS, FLT1, and PDGF-*β*R. FLT3 plays a key role in the control of hematopoiesis. High levels of WT FLT3-expression have been observed in various hematological malignancies including the majority of acute myelogenous leukemias (AMLs) and B-cell acute lymphoblastic leukemias (ALLs). In line with these findings, FLT3 is clearly expressed in the majority of leukemia cell lines and, particularly, in monocytic cell lines. In addition to these frequent abnormalities, in 1996 it was discovered that in AMLs FLT3 is frequently mutated; in fact, internal tandem duplications (ITDs) within the juxtamembrane domain of FLT3 have been reported in about 25% of patients, making it one of the most single frequent mutations in adult AMLs [1].

Subsequently, it was shown that these FLT3-ITD mutations resulted in an uncontrolled receptor activation, characterized by ligand-independent receptor dimerization, constitutive FLT3 signaling with consequent activation of STAT5 and of the RAS/MAPK and PI3 K pathways.

In 2001, another frequent class of FLT3 mutations causing constitutive receptor activation occurred at the level of the activation loop of the second kinase domain [2]; this group of mutations is represented by various abnormalities, such as substitutions, small deletions, or insertions mainly occurring at codons 835 and 836 and is detected in about 5–10% of AMLs [2].

Finally, more recently, a group of point mutations within the juxtamembrane domain of FLT3 have been described in about 1% of AML patients; these mutations involve various amino acid residues, such as 579, 590, 591, and 594 [3, 4].

As it will be discussed in detail below, several studies have suggested that the presence of FLT3-ITD mutations in AMLs was associated with an increased risk of relapse and shorter overall survival. Furthermore, it was shown that low/absent expression of the normal FLT3 allele was associated with a negative outcome.

The scope of this review is to briefly outline recent evidences indicating that FLT3-ITD mutations are leukemic driver mutations and, therefore, represent important therapeutic targets. In addition, several recent molecular studies have clearly identified subsets of AML patients, whose prognosis is considerably worsened by FLT3-ITD mutations and could considerably benefit from an efficacious FLT3 pharmacological targeting.

## 2. FLT3 and Its Ligand

The Fms-like tyrosine kinase 3 (FLT3) is a tyrosine kinase that is expressed in CD34^+^ hematopoietic stem/progenitor cells (HSCs/HPCs) and is important for both normal lymphoid and myeloid differentiation. Particularly, the FLT3 is a type III receptor tyrosine kinase belonging to the same subfamily as c-kit, M-CSF, and PDGF receptors. This receptor was cloned in 1991 by two separate groups and it was reported its expression in undifferentiated stem/progenitor cells and its absent expression on the majority of differentiated hemopoietic cells. Human FLT3 is a single peptide composed by 993 amino acids, with a molecular weight of about 155–160 KDa of the mature fully glycosylated protein.

The knockout of FLT3 gene in mice elicited the generation of viable and fertile mice, exhibiting hematological abnormalities with reduced B-cell precursors in the bone marrow and with a defect in the generation of dendritic cells in lymphoid organs [5, 6].

The ligand of Flt3, FLT3 ligand (FL), is a homodimer existing both as a membrane-bound or a soluble protein. *In vitro* studies in human hematopoietic cells have shown that this cytokine acts as a multipotent growth factor allowing the expansion of multiple cell lineages. Mice lacking FL have a deficient hematopoiesis with a reduced cellularity at the level of hematolymphopoietic organs, a reduced number of bone marrow myeloid and lymphoid progenitors, a marked deficiency of natural killer cells in the spleen, and reduced numbers of both lymphoid-related and myeloid-related dendritic cells [7].

The gene knockout studies have failed to show the effect of FLT3 loss on myelopoiesis. However, this lack of effect could be due to cytokine signaling redundancy. Some studies have shown a role for FLT3 in the control of myelopoiesis. In fact, it was shown that a part of granulomonocyte progenitors possess surface FLT3 receptors. This progenitor subpopulation was identified as early GM progenitors. A higher FLT3 expression was clearly observed in early GM progenitors than in late GM progenitors [8]. In line with this observation, it was found that early GM progenitors are decreased in FL knockout mice [8]. In line with these findings, Gabbianelli and coworkers have previously reported that FLT3 mRNA expression is very rapidly lost during erythroid differentiation, while it remained sustained during all the early stages of GM differentiation of purified human hematopoietic progenitor cells [9]. Furthermore, FL greatly potentiates the proliferation and the expansion of monocytic progenitors/precursors purified from human bone marrow/peripheral blood [9].

The study of FLT3 at the level of human hematopoietic stem cells and various progenitor subpopulations provided evidence that this receptor is expressed on repopulating HSCs, as well as in early lymphoid progenitors and in the common myeloid progenitor and in the granulocyte/macrophage progenitor [10]. FLT3 expression and signaling is required for the survival of these stem/progenitor cells [10]. The studies carried out in mice have led to conclusions slightly different from those reached in human studies. In fact, through a lineage tracing mouse model, it was shown that hematopoietic stem cell origin and maintenance does not involve a FLT3^+^ stage, while differentiation of these cells into all hematopoietic lineages involves FLT3^+^, non-self-renewing, and multipotent hematopoietic progenitor cell [11]. In line with this finding, Li and coworkers have demonstrated the existence of a common myelolymphoid progenitor FLT3^+^CD150^−^ in mice capable of generating both myeloid and B/T lymphocytes [12].

## 3. The Molecular Classification of Acute Myeloid Leukemias

The progress of our understanding of the molecular basis of AMLs is essential not only for a better understanding of the pathogenesis of this heterogeneous group of diseases, but also for the stratification of these tumors into different prognostic groups and, importantly, for the identification of new targets for development of specific therapies. The enormous progress achieved in this area during the latest years was reflected into two different classifications of AMLs according to either cytogenetic or molecular abnormalities.

The karyotype analysis is routinely performed by chromosome banding analysis and allowed to classify these leukemias into various groups of different prognostic impact. The group of favorable outcome AMLs is represented by patients with acute promyelocytic leukemia (APL) with the t(15;17)(q22;q21) PML-RARA. The group of relatively favorable outcome is represented by patients with core binding factor (CBF) leukemias with t(8;21)(q22;q22)/RUNX1/RUNX1T1 or inv(16)(p13q22)/t(16;16)(p13;q22)/CBFB-MYH11. The group with poor prognosis is associated with a group of chromosome abnormalities represented by abnormalities of 3q(abn(3q)), deletions of 5q(del(5q)), and monosomies of chromosome 5 and/or 7 (-5/-/), -17/abn17p, inv(3)(q21q26)/t(3;3)(q21;q26), or complex karyotypes. In addition to these more frequent cytogenetic abnormalities, about 20% of AML patients display rare cytogenetic abnormalities. Finally, about 40–45% of AML patients exhibit a normal karyotype at diagnosis, and therefore, this heterogeneous group cannot be further subdivided according to cytogenetic criteria.

In 2010, an international working group, including many of the major world experts of AMLs proposed a new classification system for AMLs, involving both cytogenetic and molecular criteria and allowing the identification of four genetic groups of AML patients. (A) Favorable, including patients with (15;17)(q22;q21), PML-RARA; inv(16)(p13;1q22) or t(16;16)(p13;q22), CBFB-MYH11; t(8;21)(q22;q22), RUNX1-RUNX1T1; mutated NPM1 without FLT3-ITD (normal karyotype), mutated CEBPA (normal karyotype). (B) Intermediate-I, including various types of patients all with normal karyotype and with mutated NPM1 and FLT3-ITD; wild-type NPM1 and FLT3-ITD and wild-type NPM1 without FLT3-ITD. (C) Intermediate-II, including cytogenetic abnormalities not classified as favorable or adverse; t(9;11)(q22;q23), MLLT3-MLL. (D) Adverse, including various types of cytogenetic abnormalities: -5 or del(5q); -7; abnl (17p); t(v;11)(v;q23), MLL-rearranged; t(6; 9)(q23; q34), DEK_NUP214; inv(3)(q21;q26;2); t(3;3)(q21;q26;2), RNP1-EVI1 [13]. The prognostic significance of the European Leukemia Net standardization system for reporting cytogenetic and molecular alteration in adults with AMLs was directly supported by clinical studies showing that the favorable group had the best and the adverse group the worst complete remission and disease-free survival; both intermediate groups had significantly worse outcomes than the favorable, but better than the adverse group [14].

Recently, an alternative classification was proposed entirely based on molecular biomarkers derived from the analysis of a battery of molecular abnormalities represented by the fusion genes CBFB-MY11, RUNX1-RUNX1T1, and PML-RARA, the rearranged genes *FLT3-ITD* and *MLL-PTD*, and the mutated genes *NPM1, CEBPA, RUNX1, ASXL1,* and *TP53* [15]. According to molecular and clinical data, 5 subgroups have been identified: (A) very favorable subgroup involving either PML-RARA rearrangement or CEBPA double mutations; (B) favorable subgroup, represented by RUNX1/RUNXT1, CBFB-MYH11, and NPM1 mutant, negative for FLT-ITD mutations; (C) intermediate subgroup, involving different types of abnormalities, such as CEBPA single-mutated, FLT3-ITD^+^, NPM1 mutant/FLT3-ITD^+^ and wild-type cases; (D) unfavorable group comprising MLL-PTD and/or RUNX1 mutation and/or ASXL1 mutation; (E) very unfavorable group involving patients with TP53-mutated AMLs [15]. It is important to note that the groups B, C, and D are those quantitatively more numerous [15].

Many of the mutations reported in AML, when exist alone, are not sufficient to cause the development of an acute leukemic process. This is because at least two different types of mutations are required for the development of leukemia. The mutations occurring in AMLs are subdivided into three classes: class I mutations promote cell proliferation (examples of these mutations are FLT3, c-KIT, NRAS, and KRAS mutations); class II mutations inhibit cell differentiation (examples of these mutations are CEBPA, NPM1, CBF*β*, PU1, MLL, and RARA mutations); class III mutations promote epigenetic modifications (examples of these mutations are IDH 1/2, TET2, DNMT3A, and ASXL1 mutations, i.e., mutations at the level of DNA methylation-regulating “epigenetic modifying genes”); class IV mutations at the level of tumor suppressors (examples of these mutations are TP53 and WT1 mutations). 

Although the demonstration that cellular subclones harboring several different types of mutations are observed in acute lymphoid leukemias at diagnosis was carefully documented [16, 17], some studies suggest also a consistent clonal heterogeneity of primary AMLs [18]. The study of the evolution of founding and relapse clones and analyzing the mutations in relapsed AML that existed at diagnosis and that were newly acquired during relapse provide essential information to understand the dynamic of leukemia progression during relapse, the possible selective impact of therapy, and the functional role of some mutational events. This type of analysis was recently reported in various AML subtypes providing very important information about the molecular mechanisms of disease progression. Thus, Ding and coworkers sequenced the primary leukemia and relapse genomes from eight AML patients allowing to define two major clonal evolution patterns during AML relapse: (i) the clonal evolution of the leukemias is related to the founding clone through the gain of mutations, allowing its transformation into the relapse clone; (ii) the clonal evolution of the tumor is dependent upon a subclone minoritary at diagnosis, surviving to the induction therapy, acquiring new mutations, and expanding at relapse [18]. In t(8; 21) AMLs, a minoritary subpopulation of leukemia cells that harbored c-kit mutations at diagnosis becomes dominant at relapse [19]. Furthermore, a study carried out on a small subset of CD34^+^ AML patients, mostly with normal karyotype, showed that these AMLs harbor an oligoclonal immature compartment at presentation, with the presence of a minor subpopulation in initial diagnosis samples, but dominating the bulk leukemic blasts at relapse [20].

The analysis of 28 AML pairs from patients who achieved complete remission with a chemotherapy treatment and subsequently relapsed allowed to provide evidence that incomplete eradication of AML founder clones rather than the emergence of unrelated novel clones underlies AML relapse [21].

The development of these molecular studies was essential, not only to provide a molecular classification of AMLs, but also to connect some major molecular events in a pathogenetic context, distinguishing initiating events from cooperating events [22]. Thus, according to numerous studies it becomes evident that few genetic mutations are required for the development of an AML. The first step in the leukemogenetic process is the acquisition of an initiation mutation, a driver mutation (such as AML1-ETO, PML-RAR alpha, or NPM1) occurring at the level of a stem/progenitor cell. It is important to consider that this cell already possess hundreds of mutations, “passenger,” random mutations accumulated over time. This initial driver mutation confers a growth/survival advantage to this clone that progressively expands over time. This initial leukemic clone englobes the random mutations already present in this initial cell and are propagated during clone expansion. Following the acquisition of new driver mutations, such as FLT3-ITD, the expanding clone further acquires a growth advantage and englobes in its genome the new passenger mutations developed since the development of the initial driver mutation. These clonally expanded cells may represent the “founding clone,” detected at disease presentation and containing few driver mutations and many passenger mutations. The acquisition in the time of new driver mutations further will increase the gene mutational complexity of the tumor cells due to the acquisition both of the new driver mutation(s) and to the “passenger” mutations developed in this lapse of time. The new mutational events may led to a subclonal outgrowth, adding cellular and molecular complexity to the tumor cell population.

The study of six normal karyotype AMLs with FLT3-ITD mutations allowed to propose a clonal model of evolution of these leukemias and to propose the timing of acquisition of these mutations during the natural history of development of the leukemic process. To directly test this evolution model, Jan and coworkers have sequenced the exomes of AML cells and of the residual nonleukemic hematopoietic stem cells isolated from the same patients. The purified residual hematopoietic stem cells were screened for the presence of leukemia-associated mutations and were found to possess some leukemia-associated mutations, such as TET2, NPM1, and SMC1A; however, the FLT3-ITD mutation was not found in these residual hematopoietic stem cells [23]. Considering the total number of mutations (51 mutations) present in leukemic cells, a large part of them (32 mutations) were detectable in nonleukemic hematopoietic stem cells [19]. According to these findings, the residual hematopoietic stem cells have been defined as “preleukemic” stem cells [23]. Importantly, 7 of 13 mutations in genes currently mutated in AMLs were also present in the residual hematopoietic stem cells, with the notable exception of FLT3-ITD [23]. These observations strongly suggest that in these AMLs FLT3-ITD is a secondary mutation [23]. The analysis of the *in vivo* engraftment capacity, coupled with the *in vitro* analysis at the level of single cells, allowed to show that 3/5 of these AMLs follow a pattern of clonal evolution, with later more genetically altered clones becoming increasingly dominant and more frequent than early, less altered clones [23]; however, the other two AML cases did not follow this classic evolution model in that less genetically altered clones are more expanded than descendent clones bearing additional mutations [23]. 

## 4. Is the FLT3-ITD Mutation a “Driver Mutation” or a Simple “Passenger Mutation”?

The lack of significant clinical activity of FLT3 kinase inhibitors in some clinical studies has generated the idea that FLT3-ITD could simply represent a “passenger mutation.” However, recent studies based on the use of a novel FLT3 kinase inhibitor, AC220 (also known as quizartinib), have provided strong evidence that FLT3-ITD represents a “driver mutation.” In fact, in a recent interim analysis of 53 relapsed/refractory FLT3-ITD^+^ patients enrolled in a phase II trial based on AC220 monotherapy, 45% of these patients exhibited a composite complete remission with <5% bone marrow leukemic blasts. This observation provided a unique opportunity to use the clinical activity of AC220 as a tool to try to determine whether FLT3-ITD acts as a driver or a passenger mutation. Smith and coworkers explored the possible occurrence of drug-resistant kinase mutations in FLT3-ITD in 8 patients' relapse, in spite of an initial response and the continuous treatment with AC220 [24]. Using an *in vitro* saturation mutagenesis assay, they first determined three mutations at the level of the kinase domain of FLT3-ITD, conferring resistance to AC220 [24]. The screening of the 8 patients developing resistance to AC220 showed in all these patients mutations at the level of the kinase domain [24]. These observations suggest that both FLT3-ITD and drug-resistant FLT3 kinase domain mutations occur in a leukemia-initiating cell population and represent driver mutations [24].

Comparable observations have been recently reported in a cohort of patients with FLT3-ITD^+^ AML refractory to chemotherapy and treated with the multikinase inhibitor sorafenib [25]. 12/13 AML patients treated with sorafenib showed a rapid remarkable effect due to the clearance of bone marrow myeloblasts, associated to induction of differentiation of leukemic cells; however, this response was only transient and the patients relapsed, in spite of sorafenib administration [25]. The analysis of FLT3 mutational status and of the leukemia-initiating cell population allowed to define the clonal changes occurring during the development of sorafenib resistance. Particularly, six of these patients have been screened for the presence of FLT3-ITD and FLT3 D835 mutations before sorafenib treatment and after sorafenib treatment when they became resistant to this tyrosine kinase inhibitor [25]. The level of the total leukemic blast naïve cell population, FLT3-ITD, was the only mutation detectable. However, in 3/6 cases the D835 mutation was observed in the progeny of leukemic cells generated in NOD/SCID mice by leukemia-initiating cells, thus suggesting that these mutant clones are present at the level of a very minoritary cell population and therefore are not detectable when the whole blast cell population was explored [25]. In other cases, D835 mutation was detectable only in relapsing resistant cells both at the level of the whole blast cell population and at the level of leukemia-initiating cells. Interestingly, in some cases, the emergence of a D835-mutant clone in xenotransplanted FLT3-ITD cells treated in the mice with Sorafenib was observed [25].

The ensemble of these observations support the concept that FLT3-ITD mutation is a driver mutation and tyrosine kinase inhibitors favor the selection of FLT3-ITD mutants.

Another important observation suggesting that FLT3-ITD is a driver oncogenic mutation derives from the study of myelodysplastic syndromes. In fact, in a study involving 82 patients with myelodysplasia who developed over time acute myeloid leukemia, 6% of patients had FLT3-ITD mutations at the time of diagnosis of myelodysplasia, and an additional 10% of patients acquired these mutations during disease progression to acute leukemia [26]. It is of interest to note that patients with FLT3-ITD mutations progressed more rapidly to AML and exhibited also a shorter survival [26]. These observations are compatible with the assumption that FLT3-ITD is an important driver mutation in the progression of myelodysplasias to AMLs [26].

## 5. Cell Signaling of WT and Mutant FLT3

The understanding of the signaling of WT and mutated FLT3 is essential to understand the mechanisms through which this receptor contributes to leukemia development.

The various steps of normal FLT3 activation and of the consequent signaling have been in part elucidated. Upon binding of its ligand, FLT3 dimerizes and undergoes conformational change, allowing the opening of its activation loop, unmasking the ATP binding pocket. The receptor becomes autophosphorylated at the level of tyrosine residues, inducing cell proliferation and inhibiting cell apoptosis through a series of signaling pathways. More particularly, the autophosphorylation at the level of tyrosine residues creates docking sites for signaling-transducing effectors, resulting in the final activation of signaling pathways being performed through cascade reactions involving tyrosine phosphorylation and activation of various cytoplasmic molecules. The cytoplasmic domain of the receptor contains tyrosine residues that once activated through phosphorylation become able to bind initial effectors of various signaling pathways, such as the p85 subunit of the phosphatidylinositol-3-kinase (PI3 K), phospholipase C-*γ*, RasGTPase, the adaptor protein growth factor receptor-bound protein (GRB2), and Src family tyrosine kinase, resulting in the phosphorylation of these proteins [27]. These effects are then responsible for the downstream activation of the PI3 K/AKT and MAPK signaling pathways, with consequent protection from apoptosis and induction of cell proliferation.

The molecular mechanisms for the activation of the various signaling pathways by the WT FLT3 receptor were in part delineated, and this represents an essential framework to understand the abnormalities related to the unregulated signaling originated from mutated FLT3 receptors.

Particularly, MAPK pathway is activated by FLT3 through multiple ways: (i) interaction of GRB2/SOS to tyrosines 768, 955, and 969 induces ERK phosphorylation; (ii) the interaction of GRB2 and GAB2 to these tyrosine residues recruits SHP2 and results in ERK phosphorylation; (iii) mutation of SRC binding sites of FLT3 results in reduced FLT3-dependent ERK activation [28].

The mechanism of PI3 K activation by human FLT3 is different from that observed for murine FLT3; in fact, while murine FLT3 is able to directly associate with the p85 alpha subunit of PI3 K, human FLT3 is unable to bind this subunit. In contrast, human FLT3 phosphorylates GAB1 and GAB2 and, through these intermediates, activates PI3 K [29]. 

FLT3 is able to activate the phosphorylation of SRC, and this activation is mediated through binding of this mediator at the level of the tyrosine residues 589, 591, and 599 [30]. Various proteins of the SRC family are able to interact and to be activated by FLT3.

The phosphatase protein SHP2, playing a role as positive regulator of Ras in response to growth factors, is directly recruited at the level of FLT3 at the level of Y599 [31].

In addition to molecules actively involved in the cell signaling, FLT3 activation induces the binding of negative regulators of signal transduction machinery that have to limit an excessive receptor activation. In this context, it was shown that SOCS6 binds to phosphotyrosines 591 and 919 of FLT3 [32]. Functional studies indicate that SOCS6 negatively regulates FLT3 activation, the downstream ERK pathway, and cell proliferation [32].

Many studies have investigated the quantitative and qualitative differences in the signal transduction pathways of the FLT3-ITD mutant compared to the WT FLT3. In this context, the most relevant qualitative difference consists in the capacity of FLT3-ITD, but not WT FLT3 to act as a potent STAT5 activator. *In vitro* mapping of tyrosine autophosphorylation sites led to the identification of two putative STAT5 SH2 phosphorylation sites, Y589 and Y591, located at the level of the juxtamembrane domain [33]. This finding, directly supported by mutational studies, indicated that the two tyrosine residues Y589 and Y591 are occult binding sites, unmasked by the disruption of the juxtamembrane structure by ITD [33]. The constitutively activated form of STAT5 promotes cell survival and growth through the regulation of genes involved in the cell cycle and apoptosis, such as BCL2L1, PIM1, CDKN1A, and CCND1. Particularly relevant for the biology of leukemic cells are the effects on the expression of some proteins that protect from apoptosis. In this context, particularly interesting was a study performed by Yoshimoto and coworkers [33] showing that the antiapoptotic protein myeloid cell leukemia-1 (MCL-1) is overexpressed in the CD34^+^/CD38^−^ fraction of FLT3-ITD AMLs [33]. This cell fraction is enriched for cells capable of initiating and to maintaining the leukemic process, the so-called leukemic stem cells [33]; in these cells tyrosine kinase inhibitors suppress MCL-1 expression and induced apoptosis that was reversed by the enforced MCL-1 expression [33].

The concept that FLT3-ITD-mediated STAT5 activation plays a relevant role in sustaining the survival and expansion of leukemic stem cells is supported also by additional observations. In fact, the study of mouse models of leukemia has shown that the fusion oncogene MIZ-TIF2 causes leukemia in mice, but only after a long period of latency; coexpression of FLT3-ITD confers growth factor independency for survival and proliferation, shortens disease latency, and results in an increase in leukemic stem cells [34]. STAT5 was shown to be entirely responsible for this cooperative effect between FLT3-ITD and the fusion oncogene MIZ-TIF2 [34].

It is important to note that STAT5 is directly phosphorylated by FLT3-ITD, independently of JAK2 and SRC kinases [35].

FLT3-ITD, at variance with normal FLT3, can be activated at the level of two cellular compartments, activating different signaling pathways; in fact, FLT3-ITD aberrantly activated at the level of endoplasmic reticulum phosphorylates STAT5, while the pool of plasma membrane-bound FLT3-ITD activates the MAPK and AKT signaling pathways [36].

Surprisingly, SOCS1 is one of the genes induced by aberrant STAT5 activation mediated by FLT3-ITD [37]. This finding is intriguing because SOCS1 possesses an inhibitory activity on cytokine receptor-mediated signaling. However, SOCS1 activation, while it inhibited cell signaling, originated from inhibitory cytokines, such as interferons [37]. Importantly, SOCS1 cooperated with FLT3-ITD in murine leukemia models in inducing a myeloproliferative disease [37]. These observations suggest that SOCS1 acts as a conditional oncogene [37]. In line with these observations, constitutive STAT5 activation was frequently observed in AMLs. High expression of pSTAT5 in AMLs is associated with FLT3-ITD mutations [38].

Given this central role of STAT5 activation in mediating the oncogenic effects of FLT3-ITD, it is expected that molecules inhibiting STAT5 may inhibit the survival and the proliferation of FLT3-ITD^+^ AML cells. In line with this expectancy, *primoride*, a STAT5 inhibitor displayed both *in vitro* and *in vivo* efficacy in models of AML driven by FLT3-ITD mutations [39]. Furthermore, this STAT5 inhibitor synergized with tyrosine kinase inhibitors in the induction of apoptosis of FLT3-ITD AML cells [39].

As mentioned above, SRC and other members of the SRC family are bound and activated by WT FLT3. A recent study showed that SRC is bound by the constitutively activated FLT3-ITD at the level of tyrosine residues 589 and 591; SRC binding and phosphorylation are required for subsequent STAT5 activation [40]. The phosphorylation of Y589 and Y591 in FLT3-TKD is markedly weaker than the corresponding phosphorylation observed at the level of FLT3-ITD; consequently, this FLT3 mutant scarcely activates SRC and STAT5 [40]. These observations suggest that SRC inhibition may represent a potentially important target in the treatment of FLT3-ITD^+^ AMLs [40].

Both FLT3-ITD and the wild-type receptor are able to bind Lnk, an adaptor protein that plays an important role in the control of hematopoiesis. This protein is a negative regulator of cell signaling. Lnk binds at the level of the tyrosine residues Y572, 591, and 919 of the receptor and acts as a negative regulator of FLT3-mediated ERK activation [41]. Lnk levels are low in FLT3-ITD^+^ AML cells; and overexpression of Lnk in these cells induces an inhibitory effect on ERK activation and cell proliferation [41].

Cell signaling originated by FLT3, as well as by other receptor tyrosine kinases, is regulated by phosphatases, that prevent ligand-independent receptor activation and, thus, contribute to modulation and termination of ligand-induced cell signaling. Recent studies indicate that FLT3-ITD possesses not only the property of constitutively activating cell signaling pathways, but also the capacity of inhibiting phosphatases that could inhibit its signaling. Density-enhanced phosphatase-1 (DEP-1) acts as a negative regulator of WT FLT3 autophosphorylation and signaling [42]. The studies carried out in FLT3-ITD^+^ AML cells have shown that the DEP-1 protein is expressed in these cells, but was unable to function as an inhibitor of FLT3-ITD signaling; this is due to the high ROS levels in FLT3-ITD^+^ cells leading to partial inactivation of DEP-1 by reversible oxidation [42]. Blocking ROS production restored DEP-1 activity and reduced the oncogenic activity of FLT3-ITD [42].

A recent study provided evidence about a new additional mechanism of control of WT and FLT3-ITD activation, exerted through the interaction with Axl, another receptor tyrosine kinase (RTK). This RTK, overexpressed in the majority of AMLs, is required for the activation of receptors such as c-kit and FLT3. Axl is found to target FLT3-ITD; abrogation of Axl activation diminishes the constitutive phosphorylation of FLT3-ITD and induces inhibition of cell proliferation and induction of cell death of FLT3-ITD^+^ AML cells [43]. Therefore, Axl seems to be a potentially important target to try to inhibit the signaling activity of FLT3-ITD [43].

## 6. Animal Models to Evaluate the Oncogenic Potential and the Function of FLT3 Mutations

Animal models are of fundamental importance to understand the oncogenic potential, the cellular targets, and the molecular mechanisms of potentially oncogenic molecules. Therefore, it is not surprising that a considerable effort was performed to try to develop animal models based on the enforced expression of FLT3-ITD into various types of murine hematopoietic cells and on the analysis of the consequent effects. In this context, an initial study carried out by Kelly and coworkers in 2002 provided evidence that transformation of primary hematopoietic cells and their subsequent transplantation induced an oligoclonal myeloproliferative disorder in mice, characterized by splenomegaly and leukocytosis [44]. These initial observations indicated that FLT3-ITD mutant is sufficient to induce a myeloproliferative disorder, but it was unable to develop an acute leukemia recapitulating the phenotypic features observed in humans [44]. It was, therefore, suggested that additional mutations are required to impair hematopoietic differentiation and to induce a cellular phenotype comparable to that of the human disease [44].

In 2005, Lee and coworkers developed a transgenic mouse model expressing the FLT3-ITD gene under the control of the vav-hematopoietic promoter [45]. The transgenic mice developed a myeloproliferative disease resembling thrombocythemia, without leukocytosis [45].

After some years, a more robust animal model was developed based on the introduction of an ITD mutation into the endogenous murine FLT3 locus [46]. This model, compared to previous models above mentioned, had the great advantage of avoiding exogenous promoters. These mice developed a myelomonocytic myeloproliferative disorder characterized by bone marrow expansion of myelomonocytic elements, observed both in FLT3-ITD heterozygous and homozygous mice [46]. In line with this observation, it was found that about 3% of patients with chronic myelomonocytic leukemias display FLT3-ITD mutations [46]. Although this mouse model did not allow to reproduce the AML phenotype observed in patients with FLT3-ITD mutations, it offered the opportunity to study the consequences of constitutive signaling by FLT3 on hematopoietic progenitor proliferation and differentiation [46].

Another knock-in mouse model of FLT3-ITD-related disease was developed by Li et al., who generated a mouse model based on the insertion of an ITD mutation into the juxtamembrane domain of FLT3 [47]. These mice developed a myeloproliferative disease characterized by leukocytosis, splenomegaly, and myeloid hypercellularity; at the level of bone marrow these mice displayed an increase of myeloid and dendritic cells, associated to a decrease of B-lymphocytes [47]. However, these mice did not display acute leukemia [47]. At the progenitor level, an enhanced potential to generate myeloid colonies was observed [47].

According to all these observations, it was suggested that additional cooperative events were required in addition to FLT3-ITD to progress to acute leukemia.

In these studies of double genetic hits, two potentially cooperating genetic mutations have been combined. In this context, in some studies the possible cooperation between FLT3-ITD and NUP98-HOXD13 was investigated. NUP98 (Nucleoporin 98 gene) is reportedly fused to various partner genes, including some homeobox (HOX) genes, in hematological malignancies, (mostly AMLs) with 11p15 translocations [48]. In these patients, a very high rate of FLT3-ITD (56%) mutations, mutually exclusive with RAS mutations, has been detected [48]. Previous studies, based on a transgenic mouse model in which the fusion NUP98-HOXD13 gene was expressed under the control of hematopoietic-specific vav promoter, have shown the development of a neoplastic disease with a myelodysplasia phenotype [49]. However, mice expressing both the FLT3-ITD and NUP98-HOXD13 developed AML with a short latency and with 100% penetrance [50]. These leukemias can be treated with FLT3 kinase inhibitors, thus supporting a major role of mutated FLT3 in their development; furthermore, they showed also the spontaneous loss of the WT FLT3 allele, resembling the phenomenon of loss of heterozygosity observed in spontaneously occurring human leukemias [50].

Another model of double knock-in mouse is based on the coexpression of FLT3 and MLL mutants. As mentioned above, a part of AMLs, associated with a negative prognosis, displays a mutation of the MLL gene, consisting in partial tandem duplication (MLL-PTD); as a consequence of this mutation, the MLL gene is disrupted. About 25% of AMLs with rearranged MLL gene display also FLT3-ITD mutations. A recently reported model of murine MLL-PTD knock-in showed that transgene expression in the hematopoietic compartment induced a series of abnormalities at the level of the hematopoietic stem/progenitor cells, but was unable to induce the development of acute leukemia [51]. In contrast to the single knock-in mice, the double knock-in mice for MLL-PTD heterozygous and FLT3-ITD heterozygous developed AML with 100% penetrance and with 49 weeks of latency [52]. However, if the double knock-in is developed starting from MLL-PTD heterozygous and FLT3-ITD homozygous, the time of latency for leukemia development is markedly decreased, thus highlighting the importance of the FLT3-ITD dosage [52]. 

Other studies have shown that FLT3-ITD mutations are able to cooperate with CEBPA mutations to induce acute leukemia in a mouse model; particularly, it was shown that coexpression of FLT3-ITD together with a C-terminal CEBPA mutant considerably shortened the latency period of leukemia development and markedly increased leukocytosis [53]. In another study, it was shown that FLT3-ITD considerably shortens latency of AML development in mice with biallelic CEBPA mutations [54]. The analysis of the mechanisms of cooperation between FLT3-ITD and CEBPA mutants at the level of stem/progenitor cells showed that hematopoietic stem cells and hematopoietic progenitor cell expansion elicited by CEBPA mutations is amplified by FLT3-ITD; furthermore, it was shown that in this mouse leukemia model cells with leukemia initiating capacity reside at the level of the committed myeloid progenitor compartment [54].

Other studies were devoted to analyze the cooperation between PML-RARA and FLT3-ITD in promoting the development of acute promyelocytic leukemia (APL). Appropriate mouse models of APL have provided evidence that PML-RARA expression was sufficient to promote hematopoietic progenitor self-renewal, inducing the expansion of a population of cells that are susceptible of acquiring secondary mutations, necessary for the progression to acute leukemia [55]. One of the mutations cooperating with PML-RARA in inducing leukemia could be represented by FLT3-ITD. In line with this hypothesis, Kelly and coworkers have demonstrated that retroviral transduction of FLT3-ITD into bone marrow cells obtained from PML-RARA transgenic mice resulted in an APL-like disease developing with a short latency [56]. The cooperative effect of FLT-ITD with PML-RARA on APL development is in part related to the induction of PIM2; PIM2 overexpression could replace FLT3-ITD in inducing APL together with PML-RARA [57].

In conclusion, the animal models have been of fundamental importance in defining the oncogenic activity of FLT3-ITD mutations in the context of various genetic models of AML in mice, particularly for: (a) demonstrating that FLT3-ITD alone does not fully transform a cell to leukemia, while the combination of FLT3-ITD with other oncogenic mutations can lead to the development of a full-blown leukemia in mice; (b) elucidating the molecular mechanisms underlying deregulated growth induced by FLT3 mutants; (c) developing robust *in vivo* platforms for discovery of new FLT3 inhibitors.

## 7. FLT3-ITD and Leukemic Stem Cells

Several observations suggest a functional link between FLT3-ITD and leukemic stem cells (LSCs). First, the analysis of primary FLT3-ITD^+^ AMLs showed that the CD34^+^/CD38^−^ fraction of these leukemias enriched in cells with the property of LSCs, containing the FLT3-ITD mutation [58]. Particularly, the FLT3 mutant/WT ratio was not changed by selection of CD34^+^/CD38^−^ cells, thus implying that FLT3-ITD mutations are present at the level of LSCs [58]. The engraftment of CD34^+^/CD38^−^ cells into NOD/SCID mice was inhibited by a FLT3 tyrosine kinase inhibitor [58].

In line with these observations, enforced expression of FLT3-ITD into human hematopoietic stem/progenitor cells (CD34^+^ cells) resulted in enhanced survival, proliferation and partial blocking of cell differentiation [59].

Second, some studies aiming to evaluate the NOD/SCID repopulating activity of various AML subtypes have suggested that FLT3-ITD-positive AMLs have the highest engraftment capacity in immunodeficient mice [60]. However, this conclusion was questioned by another study reporting that the NOD-SCID-engrafting capacity of AMLs does not seem to be related at some extent with FLT3-ITD mutational status [61].

Third, in FLT3-ITD-positive AMLs initially responding to treatment and then relapsing after several months, the FLT3-ITD mutation remains detectable in relapsing AML cells, thus suggesting that this mutation has occurred in a cell resistant to chemotherapy [62].

Fourth, a recent study in a FLT3-ITD knock-in model provided evidence that the FLT3-mutant may act at the level of a hematopoietic stem cell [63]. The expression of FLT3-ITD from an endogenous promoter into a murine knock-in model resulted in the development of a myeloproliferative disorder characterized by myeloid progenitor expansion [63]. In these animals, the expansion of myeloid progenitors starts at the level of a hematopoietic stem cell compartment, with progressive replacement of these cells. The depletion of the hematopoietic stem cells and the development of the myeloproliferative disorder can be blocked by treatment with the tyrosine kinase inhibitor sorafenib [63]. The depletion of the hematopoietic stem cell compartment induced by FLT3-ITD expression may help to explain why in murine models FLT3-ITD mutations fail to induce overt leukemia development [63].

These studies are important because they suggest that FLT3 mutations do not act as early hits in the HSC compartments, but rather acquired in later stages of leukemogenesis in human AMLs.

## 8. FLT3-ITD and Cell Differentiation

As mentioned above, the main biological effects related to a constitutive and aberrant FLT3 signaling originated from FLT3-ITD are related to the induction of a survival advantage and of increased proliferation. However, another important biologic effect of FLT3-ITD consists in the induction of a block in cell differentiation, and the understanding of the molecular mechanisms underlying this effect could offer the opportunity to new therapeutic strategies. In this context, initial studies carried out in 32D myeloblastic cell line provided evidence that FLT3-ITD enforced expression resulted in an inhibition of G-CSF-driven granulocytic differentiation of these cells [64]. This effect was specific for FLT3-ITD since overexpression of WT FLT3 in these cells delayed, but not inhibited granulocytic differentiation [64]. Importantly, the tyrosine kinase inhibitor CEP-701 overcomes the differentiation block of 32D cells elicited by FLT3-ITD [64].

Subsequent studies were focused to try to understand the molecular mechanisms through which FLT3-ITD could affect myeloid cell differentiation. In this context, a first study carried out using the 32D cell model expressing FLT3-ITD has shown a suppressive effect of the mutant FLT3 on the expression myeloid transcription factors, such as PU.1 and CEBPA [65]. These findings were confirmed by Zheng and coworkers who showed also that the FLT3 inhibitor CEP-711 removed the blockade of PU.1 and CEBPA expression induced by FLT3-ITD [66]. Another mechanism through which FLT3-ITD affects CEBPA is represented by ERK-induced phosphorylation of this transcription factor; following its phosphorylation, CEBPA function is inhibited [67]. In FLT3-ITD-expressing cells, such as MV4;11, either the pharmacological inhibition of FLT3 or of ERK activity induced granulocytic differentiation and, therefore, removed the FLT3-ITD-mediated differentiation block [67]. However, only 39% of FLT3-ITD AML patients displayed ERK activation, thus suggesting that seemingly there are other mechanisms responsible for Ser 21 phosphorylation of CEBPA. Thus, it was recently reported that cyclin-dependent kinase 1 (CDK1) is an FLT3-ITD-activated kinase, which is responsible for CEBPA phosphorylation on serine 21 and the blocking of its function [68]. Importantly, inhibition of CDK1 activity relieves the differentiation block in cell lines with mutated FLT3, as well as in primary patient-derived leukemic blast samples [68]. 

Another study showed a possible role of RGS2, a regulator of G-protein signaling, in mediating the inhibitory effect of FLT3-ITD on myeloid differentiation. RGS2 expression is induced during granulocytic differentiation. In fact, it was shown that FLT3-ITD elicited a suppressive effect on RGS2 expression; the repression of the expression of RGS2 by FLT3-ITD was required to inhibit CEBP alpha expression [69]. Importantly, RGS2 enforced expression reinduced the expression of FLT3-ITD-repressed CEBPA and antagonized the FLT3-ITD-induced block of cell differentiation [67]. GADD45 was shown to be another FLT3-ITD-repressed gene [70]. The downregulation of the expression of this tumor suppressor contributes to mediate the inhibitory effect of FLT3-ITD on cell differentiation [70].

The relevant role of the block of cell differentiation in the oncogenic effects of FLT3-ITD is directly supported by a recent clinical observation made in a group of AML patients with FLT3-ITD^+^ AML and with normal karyotype treated with quizartinib (also known as AC220) [71]. Interestingly, in the BM aspirates of these patients it was observed a terminal granulocytic differentiation of leukemic blasts, in association with a clinical differentiation syndrome [71]. Patients whose blasts failed to differentiate in response to quizartinib exhibit CEBPA mutation preexisting to the treatment, suggesting a mechanism of resistance to FLT3 inhibition [71]. In line with these observations, culture of primary FLT3-ITD blasts in the presence of human bone marrow stroma and quizartinib elicited cell-cycle arrest and differentiation, but not apoptosis [71].

## 9. Prognostic Impact of FLT3-ITD Mutations on Various AML Subtypes

The prognostic impact of FLT3 mutations on AMLs has been the object of many recent studies, now providing very important criteria to the clinicians for a careful stratification of patients according to various levels of risk and then for their selection for different therapeutic approaches (Table 1).

In this context, various recent studies were very informative to assess the impact of FLT3 mutations on various AML subtypes. Thus, in a recent study carried out on a cohort of 481 AML patients the impact of FLT3 mutations on core binding factor (CBF)-AMLs, normal-karyotype (NK) AMLs, and poor-risk AMLs was evaluated [72]. Among these patients, the frequency of all FLT3 mutations was 20% in CBF-AMLs, 32% in NK-AMLs and 7.6% in poor-risk AMLs. FLT3 mutations, either FLT3-ITD or FLT3-TKD, did not have a prognostic impact on patients with AMLs pertaining to either the good-risk or the poor-risk karyotype subgroups [72]; in contrast, FLT3-ITD, but not FLT3-TKD mutations, had a prognostic impact on NK-AMLs, both for that concerns the overall survival and the event-free survival [72].

Univariate analysis of AML patients according to the most frequent genetic mutations at the level of single genes has shown that FLT3-ITD mutation and MLL-PTD were associated with reduced overall survival [72].

Univariate analysis of AML patients according to the most frequent genetic mutations at the level of single genes has shown that FLT3-ITD mutations at the level of single genes have shown that FLT3-ITD mutations represented the primary predictor of outcome in intermediate-risk AML patients (Table 1) [73]. The analysis of the additional mutational status of these AMLs allowed to define three categories of intermediate-risk mutant FLT3-ITD AMLs: a first category included patients with trisomy 8 or TET2, DNMT3A, or MLL-PTD mutations who are associated with poor prognosis (3-year survival of 14.5%); a second category, with WT CEBPA, TET2, and DNMT3A and without MLL rearrangement (3-year survival of 35%); a third category, with CEBPA mutations (3-year survival of 42%) [73]. These three risk categories can be identified also in patients with normal karyotype [73].

FLT3-ITD mutations are particularly frequent in AMLs with a normal karyotype (Table 1). In this large subgroup of AMLs, about 50% of patients have FLT3-ITD and about 50% have NPM1 point mutations. These patients are, therefore, subdivided according to the presence or not of FLT3 and NPM1 mutations. Studies carried out during the last decade have shown that not only the presence of a FLT3-ITD per se, but also the FLT-ITD/WT ratio is an important determinant [74]. In addition to the FLT3-ITD, another important prognostic determinant is represented by the insertion sites of mutated FLT3-ITD; in fact, in FLT3-ITD mutated AMLs the FLT3-ITD insertion site is a determinant of clinical outcome, with patients with insertion of FLT3-ITD in the beta-1 sheet of the tyrosine kinase domain-1, displaying a shorter event-free survival and overall survival [75]. Therefore, in some studies in which the impact of FLT3-ITD/WT ratio was evaluated, FLT3-ITD-positive patients have been subdivided into two groups: those showing a FLT3-ITD/FLT3-WT ratio comprised between 0.01 and 0.5 and those exhibiting a ratio > 0.5. Recently, a study based on the analysis of 648 AML patients with normal karyotype allowed to carefully evaluate in these groups of patients the impact of the FLT3 mRNA level according to the mutation status of NPM1 [76]. This important study showed that the significant impact of FLT3-ITD on outcome was evident only in NPM1-mutated, but not in NPM1-WT AMLs [76]. Particularly, in NPM1-mutated AMLs the effect of FLT3-ITD mRNA displayed a dose dependency; thus, patients with FLT3-WT showed the best prognosis, patients with a FLT3-ITD level between 0.01 and 0.5 poorer prognosis, and patients with a FLT3-ITD level >0.5 a worst prognosis [76]. This result was supported by the analysis of both the overall survival and the relapse-free survival [76]. According to these observations, it was concluded that FLT3-ITD mRNA level may be used as a guide for the allogeneic bone marrow transplantation in NPMI-mutated AML patients. These conclusions were confirmed in another recent study showing in the group of AML patients with normal karyotype and mutated NPM1 that there is a marked difference in the relapse risk, as well in the survival, between patients with low and high FLT3-ITD/WT ratio [77].

In NK-AMLs with nonmutated NPM1, FLT3-ITD always negatively impact outcome, independently of the FLT-3-ITD/WT ratio (Table 1) [74]. However, some NPM1 wt AMLs perform equally poorly. Some studies have attempted to identify subsets of these AML patients. Thus, a very recent study identified a subset of AML patients with normal karyotype and nonmutated NPM1 and with a stem cell signature, characterized by an heptad of transcription factors (SCL, LYL1, LMO2, GATA2, RUNX1, FLI-1, and ERG); these patients exhibit an adverse prognosis when this expression signature was associated with FLT3-ITD mutations [78]. 

As mentioned above, CEBPA mutations are seen in 5–14% of AML patients and are associated with a favorable clinical outcome. Biallelic CEBPA mutations are more frequent (about the double) than monoallelic mutations. Biallelic CEBPA mutations are not associated with NPM1 mutation and with a low rate of FLT3 mutants (about 5%). In contrast, monoallelic C/EBPA mutant patients are frequently associated with FLT3 mutations (about 40% FLT3-ITD, about 20% FLT3-TKD), NPM1 mutation (about 43%), and DNMT3A mutations (about 25%). Among these patients, FLT3 mutations were observed at very high frequency (>80%) among patients with concomitant NPM1 mutations and at a lower frequency (about 35%) among patients with nonconcomitant NPM1 mutations [79]. In adult AML patients, the presence of FLT3-ITD mutations does not have a negative prognostic effect on monoallelic and biallelic CEBPA-mutant AML patients [79]. A recent study explored a large number of AML patients with double CEBPA mutants and provided evidence that only 6% of these patients display FLT3-ITD mutations [80]. These patients have usually a favorable prognosis, not modified by the concomitant presence of FLT3-ITD mutations [80]. In these patients, only the concomitant presence of Tet2 mutations was associated with a less favorable prognosis [80]. However, a negative prognostic influence of FLT3-ITD mutations in CEBPA mutant AMLs has been reported for younger AML patients [81].

As mentioned above, about 70–75% of AMLs classified as intermediate cytogenetic risk group are associated with normal karyotype, while in the remaining 25–30% an abnormal karyotype was observed. A recent study addressed the problem of the impact of FLT3-ITD mutations into through two groups of patients providing evidence that FLT3-ITD negatively impacted the prognosis in both groups, either those with a normal or abnormal karyotype [82].

FLT3 is frequently mutated in APLs, and many studies have attempted to verify the prognostic impact of these mutations. As mentioned above, FLT3-ITD mutations are observed in 25–35% and FLT3-TDK mutations in 10–15% of APL patients. In the majority of these studies, the prognostic impact of FLT3 mutations on APL was evaluated in parallel with the white blood cell (WBC) count at diagnosis, a well known negative prognostic factor in these leukemias. In an initial study, Gale and coworkers have shown the WBC, but not the FLT3 mutations, was the sole independent prognostic factor for survival and relapse [83].

In a second study, Chillón et al. have investigated the prognostic impact of the size of FLT3-ITD duplications and the ratio FLT3-ITD/FLT3-WT, as well as of WBC counts showing that all these three parameters were independent prognostic factors in APL [84].

Barragán and coworkers have evaluated the prognostic impact of FLT3-ITD and FLT3-D835 mutations on a large set of APL patients [85]. In this study, the frequency of FLT3-ITD was 22% and of FLT3-D835 was 9% [85]. FLT3-ITD was correlated with higher WBC counts and was associated with higher relapse and lower survival rates; in contrast, FLT3-D835 mutation had no impact on prognosis and was not associated with peculiar biologic features [85]. However, Schnittger and coworkers failed to show a significant impact of the FLT3-ITD mutation status per se on prognosis of APL; however, a higher FLT3-ITD/FLT3-WT ratio (>0.5) was prognostically adverse [86].

In a more recent study, Gallagher and coworkers have evaluated the role of FLT3 mutations in the relapse of APL patients treated with ATRA and chemotherapy [87]. About 15–30% of APL patients relapsed after therapy based on ATRA and chemotherapy. A part of these relapsing patients (about 40%) harbor mutations in the ATRA-targeted ligand binding domain of PML/RAR*α* (PR*α*/LBD^+^) [87]; importantly, a significant proportion of these PR*α*/LBD^+^ patients relapsed off ATRA selection, suggesting an active role of the PR*α*/LBD^+^ mutation [86]. 37% of the relapsing patients exhibited FLT3-ITD mutations which were positively associated with PR*α*/LBD mutations in APL patients relapsing on ATRA and negatively associated with PR*α*/LBD mutations in those relapsing off ATRA [87]. In contrast, PR*α*/LBD mutations occurring off ATRA treatment positively correlated with the presence of chromosome abnormalities [87]. In addition to these findings, it was also observed that the ratio of mutant FLT3-ITD to normal FLT3-WT allele did not change in FLT3-ITD^+^ patients in the relapsing disease compared to the presentation disease; this last observation suggests that the FLT3 mutations, considered as single lesions do not contribute alone to disease relapse [87]. However, the fact that FLT3-ITD mutations are concomitant with PR*α*/LBD mutations in patients who relapse under ATRA treatment suggests an ATRA-dependent complementary action between these two mutations [87].

Recently, a new recurrent gene mutation (frameshift mutation) in the DNA methyltransferase gene DNMT3A was identified. This mutation occurs in about 20–25% of AML patients and was particularly frequent among patients with an intermediate-risk cytogenetic profile, while it was absent among patients with a favorable-risk cytogenetic profile [88]. This mutation was observed mainly in patients corresponding to the FAB M4 (myelomonocytic) and M5 (monocytic) AML subtypes [88, 89]. In about 60% of cases, the DNMT3A mutation was associated with NPM1 mutations, while in 40% of cases with FLT3 mutations. The presence of a DNMT3A mutation was associated with a worse survival among patients with a normal cytogenetic profile or with an intermediate-risk profile; when patients with FLT3-ITD mutation were classified according to the DNMT3A mutational status, those carrying a DNMT3A mutation had significantly poorer outcome than those without DNMT3A mutation [88].

Wakita and coworkers have explored the occurrence of FLT3-ITD mutations at diagnosis and at relapse in a group of 34 patients displaying in 7 patients DNMT3A mutations and in other patients other epigenetics-modifying gene mutation (4 displayed IDH1/2 mutations and 5 exhibited TET2 mutations; two patients concomitantly DNMT3A and TET2 mutations and two DNMT3A and IDH1/2 mutations; therefore, 12/34 patients had epigenetics-modifying gene mutations) [90]. At diagnosis, FLT3-ITD mutations preferentially occurred at the level of patients with epigenetics-modifying gene mutations (5/22) [79]. All the mutations at the level of the epigenetics-modifying genes were conserved at relapse; 3/11 FLT3-ITD mutations were lost at relapse, all in AML patients negative for mutations at the level of epigenetics-modifying gene mutations and two new FLT3-ITD mutations [90]. These findings were interpreted as suggestive of the hypothesis that an initial mutation at the level of an epigenetics-modifying gene promotes genetic instability, thus facilitating the occurrence of FLT3-ITD mutations and the expansion of FLT3-ITD positive clones [90].

Gonen and coworkers identified a membrane marker, CD25 (the alpha-chain of the interleukin2 receptor (IL-2R)), whose expression in part overlaps with DNMT3A mutant AMLs. In fact, these authors have shown that 13% of AMLs display CD25 expression, of whom 92% had intermediate-risk cytogenetics. The majority (>60%) of CD25^+^ AMLs display mutations at the level of epigenetics-modifying gene; furthermore, the large majority (76%) of these AMLs display FLT3-ITD mutations [91]. Importantly, when all FLT3-ITD^+^ AMLs were stratified according to CD25 expression it appeared evident that only CD25^+^/FLT3-ITD^+^ AMLs have a negative prognosis [91]. Interestingly, CD25^+^ AMLs exhibit an elevated expression of the alpha-chain of the IL-3R (CD123). CD123 was found to be overexpressed in about 40% of AML patients, and its overexpression was associated with increased cellularity and a poor outcome [92] and, frequently, with FLT3-ITD mutations [93]. The ensemble of these observations define an AML subtype with mutation of at least one epigenetics modifying gene, CD25 expression, and CD123 overexpression and associated with a poor outcome.

## 10. Conclusions

In conclusion, the studies carried out during these last years support an important role of FLT3-ITD as a driver mutation playing a relevant role in AML development. This conclusion is supported by various lines of evidence and led to identify FLT3-ITD as a valid therapeutic target for some AMLs, in large majority of cases associated to a negative prognostic index. Given the importance of FLT3-ID and of the FLT3 pathway in the pathogenesis and prognosis of patients with AML, consistent efforts are in progress for the development and clinical testing of FLT3 inhibitors (reviewed in [94]). 

## Figures and Tables

**Table 1:** Risk stratification of AMLs according to the FLT3-ITD status.

Cytogenetic classification	FLT3-ITD status	Mutations	Overall risk profile
Favorable	Negative	t(15;17) (q22;q12) PML-RARA	Favorable
Favorable	Positive (30–35% of cases)	t(15;17)(q22;q12) PML-RARA	Usually favorable, but patients with long FLT3-ITD and low PML-RARA expression have less favorable prognosis and patients with high FLT3-ITD/FLT3-WT ratio
Intermediate or normal karyotype	Negative	DNMT3A mutation	Intermediate
Intermediate or normal karyotype	Positive (40% of cases)	DNMT3A mutation	Unfavorable
Intermediate or normal karyotype	Negative	NPM1 mutation	Favorable
Intermediate or normal karyotype	Positive (30–40% of cases)	NPM1 mutation	Intermediate (low FLT3-ITD/FLT3-WT ratio), unfavorable (high FLT3-ITD/FLT3-WT ratio)
Intermediate or normal karyotype	Negative	No NPM1 mutation	Usually intermediate; unfavorable (some cases)
Intermediate or normal karyotype	Positive (15–20% of cases)	No NPM1 mutation	Intermediate; unfavorable (associated with a stemness signature)
Intermediate or normal karyotype	Negative	CEBPA double mutant	Favorable
Intermediate or normal karyotype	Positive (6% of cases)	CEBPA double mutant	Intermediate
Intermediate or normal karyotype	Negative	CEBPA single mutant	Unfavorable
Intermediate or normal karyotype	Positive (30–40% of cases)	CEBPA single mutant	Unfavorable
Intermediate or normal karyotype	Negative	MLL PTD	Unfavorable
Intermediate or normal karyotype	Positive (30–35% of cases)	MLL PTD	Unfavorable
Unfavorable	Negative	Various mutations	Unfavorable
Unfavorable	Positive (6-7% of cases)	Various mutations	Unfavorable
